# Iodine an Antidote to the Venom of the Rattle-Snake

**Published:** 1849-01

**Authors:** James Whitmire

**Affiliations:** Metamora, Ill.


### ARTICLE X.

*Iodine an Antidote to the Venom of the Rattle-Snake.* By Jas. Whitmire, M. D., of Metamora, Ill.

I wish to say to the profession, through the North-Western Medical and Surgical Journal, that I believe iodine to be an antidote to the virus of the rattle-snake, and, in fact, the whole tribe of serpents.

My opinion, as to the antidotal property of iodine, has been confirmed by many cases that I could give from my case book, in which I used the tinc. of iodine alone, with the effect of putting an entire stop to the swelling and pain, in from twelve to sixteen hours. I have used it in bites of the rattle-snake, viper, and copper-head, on both man and beast, with complete success. My manner of using it is to paint the part that is bitten, and as far as the swelling extends, with three or four coats of tinct. (pharmaceutical strength) twice daily; and should the swelling extend, which it almost always does after the first application, if made any time soon after the infliction of the wound, I follow it up with the paint. By the time the third application is made, the tumefaction will cease to extend, and three or four more applications will generally restore the limb, or part affected, to its natural state, save perhaps an obtuse sensibility to the touch, owing perhaps to the cuticle being destroyed, and some soreness of the muscles, which will remain a longer or shorter period.

A short history of my first acquaintance with this article may not be uninteresting to some of my readers. In June, 1846, I was reading a little work, by Dr. Guthrie, on the use of iodine, in enlargements of the joints, goiter, &c., where its remedial effects were ascribed to its tonic effect upon the capillary and lymphatic vessels of the part. During this time, a lad rode up to my office door, and said that his brother had been bitten by a rattle-snake, and wished me to see him immediately. I had just entered upon the duties of my profession, and, as a matter of course, to use a vulgar phrase, was stumped to know what to do for the boy. I had seen several cases of the kind, and some of them very troublesome ones, too, in which there had been used every thing that had ever been recommended, both by the profession and the old ladies. So that it was doubtful in my mind whether there was any remedy known that could be depended upon. I was satisfied that the immediate effect of the virus was a suddenly diffused, low grade of inflammation in the part in which it was injected, speedily extending its ravages until the whole system became a prey to its morbific influence; at which time, fever, parched tongue, delirium, &c., followed in the train. The immediate contact of the virus with the capillary and lymphatic vessels of the part is no doubt the cause of the tumefaction that immediately comes on; the virus destroying natural tone. Either the above is true, or the swelling is produced upon the principle of *ubi irritatio ibi fluxus*. This process of reasoning led me to a trial of the tinct. of iodine.

In about two hours from the time the boy was bitten, I saw him. He had received the wound about midway between the internal malleolus and the inferior portion of the os calcis; and the swelling had already extended to within three inches of the knee. There was severe pain in the part, nausea, and occasional vomiting. I proceeded to paint the foot and leg as high as the knec with four coats of the tinct. of iodine, and directed four more coats to begin at bed-time, and repeated in the morning. If the swelling extended above the knee, it was to be followed up with the paint. I then gave my patient a dose of Hoffman's anodyne, and a pretty active dose of Epsom salts, with directions to leave the leg uncovered the whole time, and took my leave. The next day the boy came to town, on horseback, to see me. The swelling had ceased to extend about twelve o'clock in the night, and at this time had decreased very considerably. In three or four days, he experienced no inconvenience from the bite, and went about his ordinary occupation. Since that time, I have had numerous cases of the same kind, all of which have terminated equally well under the same treatment. It is my opinion, therefore, that the iodine, being absorbed, comes in contact with the virus, and neutralizes it, at the same time, giving tone to the engorged capillaries of the part, enabling them to empty themselves of their engorgement. And, if the wound has been inflicted so long that there is effused serum in the cellular tissue, from debility of the vessels, the tinct. of iodine is none the less applicable, as it will speedily promote its absorption.

*October* 24*th*, 1848.

